# Cyclin–CDK Complexes are Key Controllers of Capacitation-Dependent Actin Dynamics in Mammalian Spermatozoa

**DOI:** 10.3390/ijms20174236

**Published:** 2019-08-29

**Authors:** Nicola Bernabò, Marina Ramal-Sanchez, Luca Valbonetti, Juliana Machado-Simoes, Alessandra Ordinelli, Giulia Capacchietti, Angela Taraschi, Barbara Barboni

**Affiliations:** 1 Faculty of Bioscience and Technology for Food, Agriculture and Environment, University of Teramo, Via Renato Balzarini 1, 64100 Teramo, Italy; 2 Istituto Zooprofilattico Sperimentale dell’Abruzzo e del Molise “G. Caporale”, Via Campo Boario 1, 64100 Teramo, Italy

**Keywords:** spermatozoa, cyclin–CDK complexes, actin dynamics, capacitation, acrosome reaction, biological network, computational model

## Abstract

Mammalian spermatozoa are infertile immediately after ejaculation and need to undergo a functional maturation process to acquire the competence to fertilize the female egg. During this process, called capacitation, the actin cytoskeleton dramatically changes its organization. First, actin fibers polymerize, forming a network over the anterior part of the sperm cells head, and then it rapidly depolymerizes and disappears during the exocytosis of the acrosome content (the acrosome reaction (AR)). Here, we developed a computational model representing the actin dynamics (AD) process on mature spermatozoa. In particular, we represented all the molecular events known to be involved in AD as a network of nodes linked by edges (the interactions). After the network enrichment, using an online resource (STRING), we carried out the statistical analysis on its topology, identifying the controllers of the system and validating them in an experiment of targeted versus random attack to the network. Interestingly, among them, we found that cyclin-dependent kinase (cyclin–CDK) complexes are acting as stronger controllers. This finding is of great interest since it suggests the key role that cyclin–CDK complexes could play in controlling AD during sperm capacitation, leading us to propose a new and interesting non-genomic role for these molecules.

## 1. Introduction

According to WHO, infertility is a “disease of the reproductive system defined by the failure to achieve a clinical pregnancy after 12 months or more of regular unprotected sexual intercourse” [1], and it affects around the 15−20% of couples. Among these, the male factor is responsible for approximately 50% of the cases (30% alone and 20% co-responsible with female factors [2]). There are many known causes associated with the appearance of male infertility, but in spite of the great effort carried out by researchers in recent years, 40−50% of the cases are due to still unknown causes (idiopathic male infertility [3]). This condition is characterized by a worsening of patient life conditions with medical and psychological negative consequences [4], an increase in the costs for both the couples and the National Health Services, and often by the necessity to undergo repeated cycles of medically assisted procreation (MAP). For these reasons, further research is still needed to decipher the exact molecular mechanisms related to the sperm function, in order to improve the current diagnosis tools and to develop specific treatments for men suffering from idiopathic infertility.

In this context, it is very important to consider that spermatozoa, achieve their fertilizing ability in the female oviduct, where they reside for a period varying from hours to days (depending on the species) bound to the epithelium, forming a *sperm reservoir* [5,6]. Here, they undergo a series of events collectively known as capacitation, a process involving different events, such as the cholesterol efflux from the sperm membrane [7,8], the increase of intracellular Ca^2+^ concentrations [9,10,11], the production of reactive oxygen species (ROS) [12], the activation of a bicarbonate/cAMP/PKA-dependent pathway and the subsequent proteins phosphorylation [13,14,15], and the cytoskeletal remodeling of actin fibers [16,17,18]. The latest is one of the keys events in driving the relocalization of membrane antigens involved in capacitation, in sperm-egg recognition and binding, and in the exocytosis of enzymes from the acrosome (the acrosome reaction (AR)), allowing the sperm cell to surpass the cumulus oocyte complex and the zona pellucida barriers to fertilize the egg.

Since the molecular mechanisms involved in control of actin dynamics (AD) during capacitation remains not completely understood, recently, our group carried out an in silico and in vitro study. Its results suggested that cyclin-dependent kinase (cyclin–CDK) complexes could be involved in control of this event, as demonstrated by the negative effects of a cyclin–CDK-specific inhibitor (Aminopurvalanol A) on actin polymerization with dramatic consequences on sperm capacitation and on in vitro fertilization outcome [19]. On this basis, here we developed a computational model devoted to describe the role of cyclin–CDK complexes in controlling the actin dynamic in mammalian mature spermatozoa. To this aim, in agreement with biological networks theory, we represented the molecules participating in the AD as nodes linked by directed edges (the interactions among them). Then, by the study of network topology, we inferred important information, useful to improve our knowledge on sperm physiology.

## 2. Results

### 2.1. Networks Creation, Analysis, and Visualization

**Figure 1:**
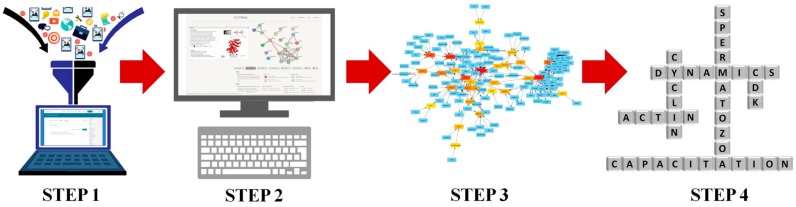
Experimental design. The figure illustrate the four step followed during the development of the work. Step 1 represents the data collection from the literature; step 2 entails the data enrichment by using specific system biology tools; step 3 implies the creation, analysis, and visualization of the network; and step 4 represents the inferences about the mechanisms controlling actin dynamics (AD).

**Figure 2:**
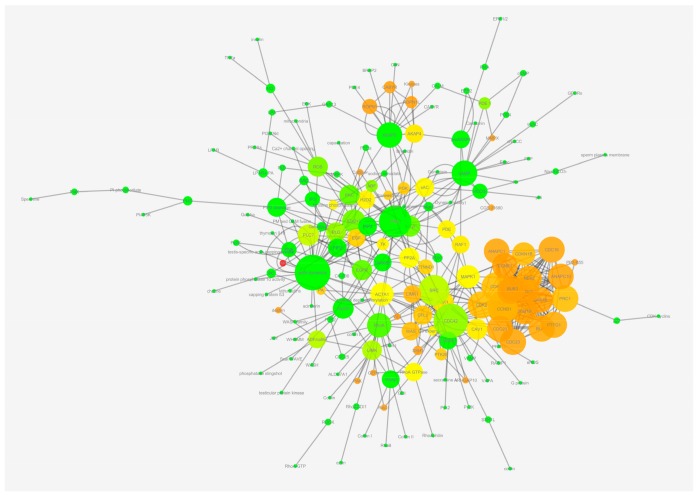
Network of AD. The network represents the AD during the post-ejaculatory life of spermatozoa. The network was created with Cytoscape 3.1.2, and the node size is proportional to the number of links per node, with the color depending on the clustering coefficient (from green = 0 to red = 1).

The experimental design, including all the steps for the creation and analysis of the network, is illustrated in Figure 1. As result of a manually-curated data analysis, we obtained a network (Data Base Network, DB Network) with 167 nodes and 274 links. After the STRING analysis of proteins involved in actin remodeling, we obtained a second network (STRING Network) with 49 nodes and 287 links. After merging the two networks, we obtained the final network (Merged Network, MN) constituted of 188 nodes and 558 links (Figure 2). The “in node degree” distribution and “out node degree” distribution follow an exponential law, characterized by a negative exponent (−1.183 and −1.314, respectively); the value of clustering coefficient is very low (0.127) and is uncorrelated with Node Degree (R^2^ = 0.003). The values of its main topological parameters are listed in Table 1 and in Supplementary Material S1.

### 2.2. Identification of Controllers within the Network

To identify the Controller active trough the network, we identified the nodes that are both hubs (i.e., the nodes with higher connectivity) and bottlenecks (i.e., the nodes that are mainly involved in control of flow within the network).

The hubs were identified based on the following formula [20]:

(1) ND>μ+σ

where:

ND = node degree

μ = averaged node degree

σ = standard deviation of node degree

They represent about the 16% of nodes (31 on 188) and are listed in Table 2.

**Figure 3:**
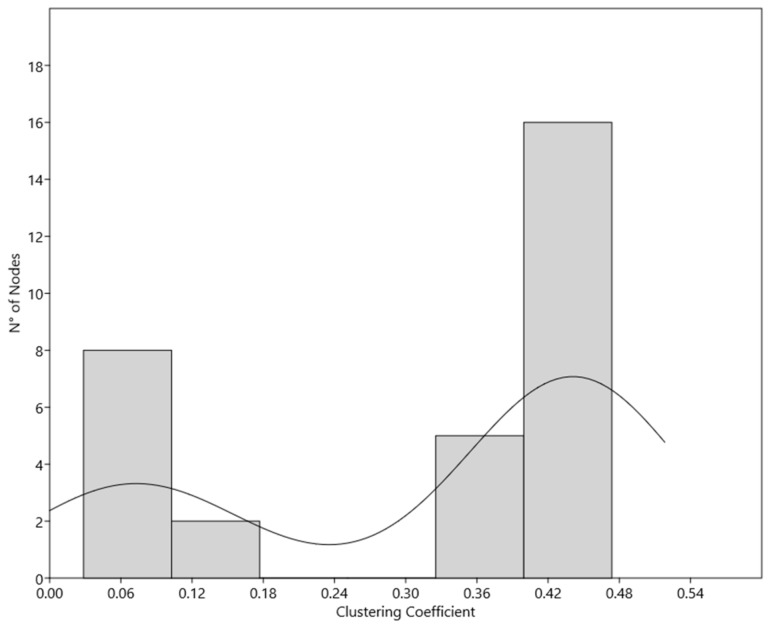
KDE analysis. Histogram shows the subpopulations in hubs based on the Clustering Coefficient value.

As expected, among the hubs, the node degree and the clustering coefficient did not correlate (*r* = −0.271, R^2^ = 0.114), while the Kernel density estiamtion (KDE) analysis (whose results are presented in Figure 3) shows the existence of two different subpopulations of hubs, characterized by different values of clustering coefficient (0−0.2 and 0.3−0.5, respectively), as shown in Table 3.

In the following step, we listed the nodes based on their values of bottleneck score and selected the first thirty-one nodes (the same number than hubs), in keeping with a previous study [20]. The list of the selected nodes is presented in Table 4.

**Figure 4:**
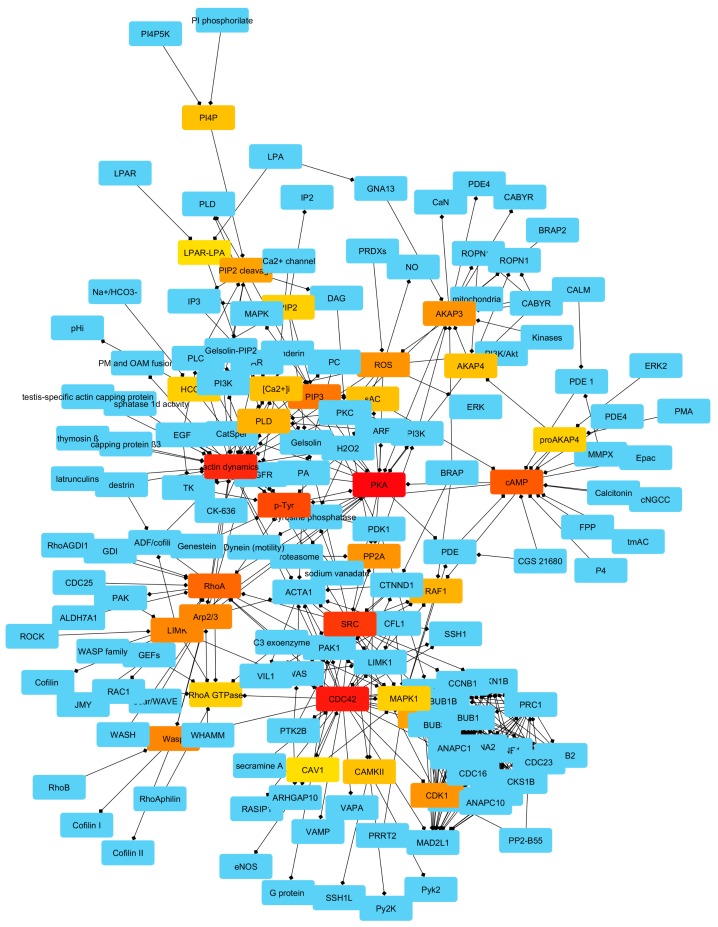
Bottlenecks network active in control of information flow within MN. The node color is depending on their bottleneck score (form light yellow for lower values to dark red for higher ones).

By using the CytoHubba plug-in, we computed the network of bottlenecks, which represents the back-bone of information flow control within MN (see Figure 4).

**Figure 5:**
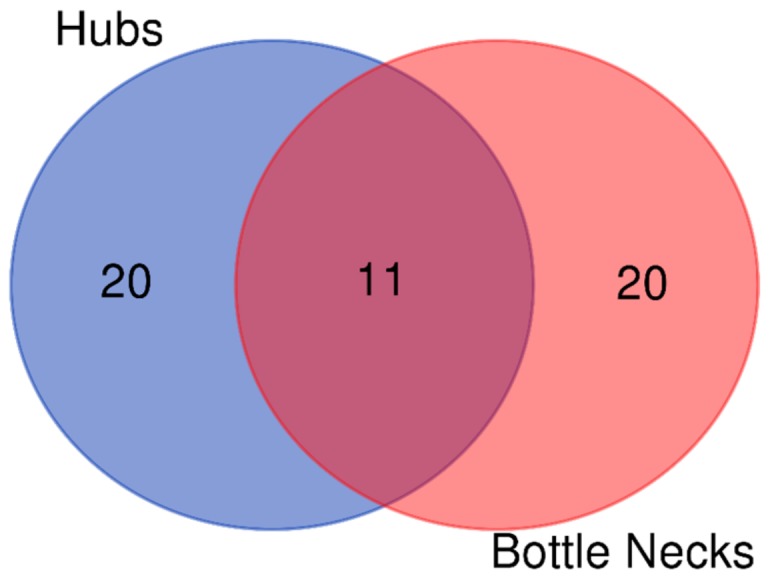
Venn diagram used to compute the nodes that are both hubs and Bottle Necks.

Finally, as computed with an online open software (http://bioinformatics.psb.ugent.be/webtools/Venn, 06/12/2019), eleven nodes (see Figure 5) were either hubs and bottlenecks, listed in Table 5.

### 2.3. Targeted vs. Random Attack to MN

**Figure 6:**
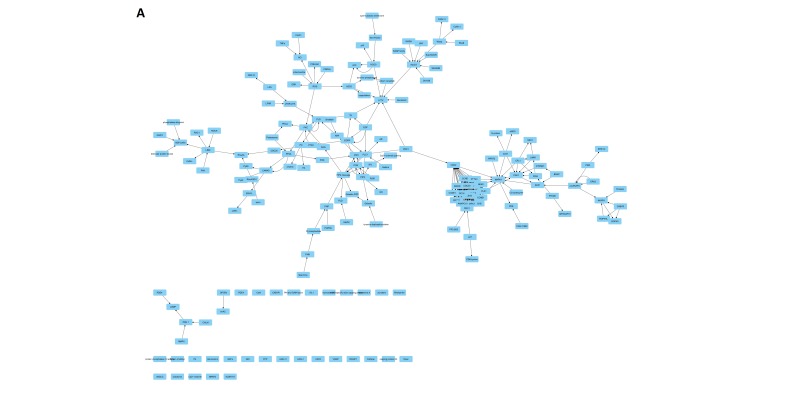

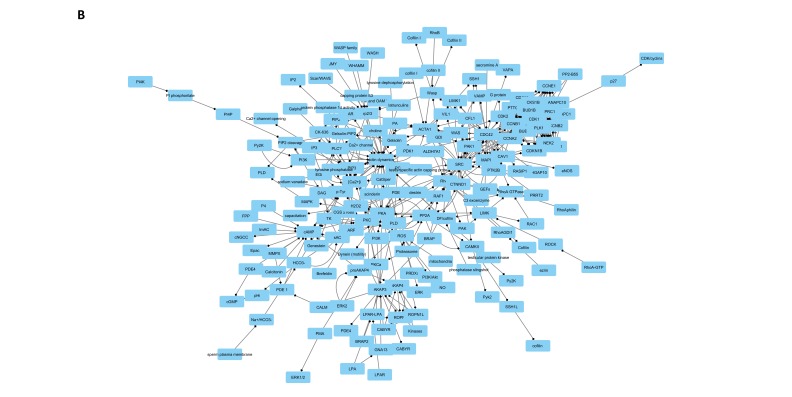
Diagram showing the effects of the removal from the network of controllers identified by our analysis (target attack), Panel **A**, compared with the effect of the removal of the same number of randomly selected nodes (random attack), Panel **B**. As it is evident, in the first case the network collapsed, in the second one no important changes in network topology are evident.

To test the reliability of our inference, we tested the effects of node removal from the networks with a computational experiment: we removed the controller (targeted removal) or different sets of 11 nodes randomly identified (function “=randbetween (min; max)” Excel 2003). In the first case, we obtained the network collapse, while in the second, we did not find evident effects in term of network stability (see Figure 6, for an exemplification).

## 3. Discussion

It is well known that mammalian spermatozoa, once ejaculated, must undergo a complex process called capacitation to become fully fertile. During capacitation several biochemical changes affect the whole sperm physiology, leading to modifications in the chemical composition of the membrane and to a deep cytoskeleton reorganization [8,21,22]. This last event has been deeply studied by different groups and in different species, with the delivering of interesting molecular data that unfortunately still remain unorganized.

Here, we adopted a computational biology approach to gather the available data in order to reconstruct the active pathway responsible for the control of AD in spermatozoa. This strategy has been recently developed and is very promising in giving important data in several branches of biology, from cancer [23] to biochemical machinery [24] to germ cells physiology [20,25,26,27]. More in detail, we used the biological networks to carry out an operation of reverse engineering in four steps: first, we collected the data from the literature (Step 1); we enriched them by using a specific systems biology tool (STRING) (Step 2); we created, analyzed, and visualized the networks (Step 3); and, finally, we took the inference about AD control mechanisms (Step 4). By performing this kind of analysis, it is possible to compute some important topological parameters.

The first one to be discussed is the number of nodes (i.e., the number of molecules involved in AD pathway). Interestingly, these results are similar to prior ones found in several others pathways involved in control of important biological events, such as input (phototransduction) and output events (muscle contraction), information processing-(neurotransmitters release cycle) and energy metabolism-related pathways (ATP, glucose metabolism), endocrine control (insulin), cells cycle regulators (p53, pRb and c-Kit), and whole body coordination events (circadian clock) [28]. This leads us to speculate that this parameter could be influenced by the balance between the energetic costs of maintaining the integrity of each molecule and the need of stability within a system. Specifically, the metabolic investment to codify, synthesize, maintain, modulate, and, finally, catabolize each molecule could led to the evolution of transduction pathways characterized by the smallest possible number of molecules, in order to reduce the energy cost. At the same time, the need to ensure the highest stability for the system could favor the evolution of pathways composed by the largest number of molecules, since any fluctuation or failure of a single element will affect in a much more limited way the whole system the higher is the number of its components [28].

In addition, based on node degree distribution and its relationship with clustering coefficient, it was possible to classify the network as a scale free, in accordance with the Barabasi-Albert model. In this model, the node degree follows the general power law:

(2) $$P\left(k\right)\sim k^{-b}$$

without being correlated with the clustering coefficient. This is a very intriguing finding because this topology confers to the network biologically relevant properties. First, as it has been extensively discussed, Barabasi-Albert networks are robust against random attacks and, at the same time, vulnerable to targeted attacks (see below for the experimental validation). It is possible to explain this, considering that most of the nodes are scarcely linked. Consequently, when their function will be affected by an external perturbation, the consequences would be negligible in terms of network stability. As it has been calculated (http://networksciencebook.com/chapter/1) in scale-free networks with *γ* < 3 the breakdown threshold converges to 1. Thus, it would be necessary to remove virtually all the nodes to break the network apart. Since the actin dynamic is a crucial event in sperm cells survival and acquisition of fertilizing ability, it is a useful pattern to guarantee that it will remain unaffected by eventual external and internal perturbations, therefore increasing the probability to fertilize.

On the other side, the presence of a low number of nodes (or molecules) able to exert a great control over the whole network allows it to save energy. Thus, the whole system can be modulated with high efficiency by controlling only a small number of molecules, reducing in this way the energetic cost and facilitating or accelerating the cell response. As already noted, incidentally, the identification of a small numbers of hubs could be helpful to control the biological systems with the purpose of developing diagnostic or therapeutic strategies for infertility or male contraception [28].

In addition, due to the small values of clustering coefficient it is possible to exclude the presence of hierarchical patterns, thus avoiding loops or clusters that could slow or interfere with the propagation of messages [28,29], with the exception of cyclin–CDK system (see below). Taken together, with the easy navigability of the network, demonstrated by the characteristic path length values of about 6.8, these features confer to the network a behavior both globally and locally efficient [30]. Indeed, the small number of passages through the network minimizes the loss of information due to the signal decrease and any local perturbation could influence the whole network in a very short time. Moreover, it increases the responsiveness of spermatozoa cytoskeleton organization to intracellular and extracellular stimuli.

Looking more in detail to the network clusterization, using the Cytocluster plug-in (IPCA algorithm for detection of dense subgraph in protein-protein networks), it has been possible to identify a dense structure involving the cyclin–CDK complexes. This is a noteworthy finding since it highlights a specific feature of the system. Indeed, likely as a safety strategy, the activity of these complexes is well modulated by the balancing of several entities interacting between each other, promoting the stability of the system and avoiding problems in it functioning.

Afterwards, by adopting a double step approach, we isolated the molecules exerting a greater control within the network. In particular, we identified the hubs, finding that it is possible to recognize two different hubs subpopulation, on the basis of their clusterization. This difference could to be in agreement with the hypothesis of Han and colleagues, which defined two different types of hubs ‘party’ hubs, which interact with most of their partners simultaneously, characterized by lower values of clustering coefficient, and ‘date’ hubs, which bind their different partners at different times or locations, more clustered [22]. In our network, the subpopulation 1 could account for date or local hubs and contains nodes related to the signal transduction and, mainly elements of cyclin–CDK system. The other one, could represent the party or general hubs list, and contains nodes, such as actin, AD and the universal messengers active in modulation of virtually all the pathways, such as Ca^2+^ of cAMP.

Then, we analyzed a second class of nodes that exert a strong control: that of bottlenecks, which are mainly involved in control of information flow within the network, and we computed their network. In this case, also, elements of the cyclin–CDK complexes seem to exert an important role.

Finally, we were able to identify 11 nodes that are both hubs and bottlenecks, as key controllers of AD network. It is very important to note two findings:

I: The process of actin cytoskeleton remodeling seems to be under the control of 11 molecules. It means that about 5% of nodes (11 on 188) are able to regulate such complex and important event.

II: On 11 controllers, 2 are directly related to actin (AD and ACTA1), and on the other nine, one third (3 on 9) are belonging to cyclin–CDK system (CCNA2, CDK1, CDC42).

To test the reliability of our hypothesis we performed an experiment by comparing the consequences in term of network integrity of the removal of the 11 putative controllers with that of several sets of 11 randomly selected nodes (see Figure 6). We found that the targeted removal of selected ones caused the collapse of the network, while the removal of 11 randomly selected nodes in the most of the cases did not have detectable consequences on network topology. In our opinion, these data are very interesting, because they clarify the signal cascade involved in control of actin dynamic and allow to confirm our data [19] re-interpret our data from Amaral and co-workers [31], that indicated the presence of several pathways previously neglected in the male gamete physiology. Concretely, they pointed out the presence of cell cycle-related proteins, in particular of cyclin–CDK) complexes and suggested that these proteins might be remnants of the spermatogenesis with no relevant function in mature spermatozoa. Indeed, although with controversy [32,33,34,35,36], it is well established that mature ejaculated spermatozoa do not have nuclear transcription activity, there is no protein synthesis from nuclear-encoded genes and they are not able to activate the cell cycle. Here, on the contrary, we could hypothesize that cyclin–CDK complexes are actively involved in control of actin function through capacitation. Until now it has been proposed that the bicarbonate present in great concentrations in the oviductal fluid (or in artificial systems), may induce the activation, via a sAC/cAMP/PKA-mediated pathway, of the lipid scrambling at the plasma membrane level. While gradually the plasma membrane acquires the ability to fuse with the outer acrosome membrane (OAM) thanks to the cholesterol extraction [7,37,38,39,40,41], the G-actin polymerizes forming a network of F-actin, which acts as a diaphragm between the two membranes avoiding their premature fusion. Once the capacitation is completely achieved and the physiological stimulus (zona-pellucida proteins) are met, the induced calcium peak causes the fast depolymerization of actin structure, and the AR can occur.

In this context, the model proposed could shed some light on the underling molecular mechanisms and could open new perspectives in the study of spermatozoa signaling. In particular, the data exposed suggest that in sperm cells cyclin–CDK complexes and their related molecules could be active, with a non-genomic effect, in controlling AD. On the other hand, we know that they remain active in several other cellular models as non-genomic effectors, as it has been well reviewed by some authors [42,43].

What still remains to elucidate is the possible role of actin cytoskeleton as an effector, rather than a passive target, in the signaling systems. In fact, it has been proposed that AD could have a role as a general coordinator of several important biological events. It is well-known that polymerized actin forms a continuous and dynamic connection between virtually all the cellular structures and that it presents an enormous surface area on which proteins and other molecules can dock [44]. This datum is strengthened by the finding that the plasma membrane surface area of a 20-μm-diameter generic cell is on the order of 700 μm^2^. By contrast, the total surface area of a typical concentration of 10 mg/mL F-actin is 47,000 μm^2^ [44] and that the diffusion along the cytoskeleton could be a reliable alternative for intracellular trafficking and signaling [45]. Our group has recently proposed that, together with other well-known molecules involved in intracellular signaling, such as Ca^2+^ or ATP, AD might provide a signal transduction route and acts as a macromolecular scaffold, probably contributing in this way to the spatial organization of those signaling pathways components participating in the spermatozoa functional maturation [29].

In conclusion, the model here stated clearly shows how cyclin–CDK complexes are active in sperm signaling and in control of AD, thus opening new perspectives in the potential design of diagnostic and therapeutic strategies that could help in the approaching of male infertility.

## 4. Materials and Methods

### 4.1. Data Collection, Network Creation, and General Analysis

As the data source, we used a validated and curated database [46], which describes the molecular events occurring during the capacitation-dependent actin remodeling process [46] updated with the recent literature from an accurate search in PubMed and Google Scholar.

The freely available and diffusible molecules, such as H_2_O, CO_2_, P_i_, H^+^, and O_2_ were omitted, since they were considered not necessary and in some cases the record did not represent a single molecule but a complex event, due to the fact that most of the single molecular determinant of the phenomenon are still unknown. The quality control was realized as previously described [27]. Briefly, as a reference, two expert researchers on spermatozoa biology carried out an independent literature analysis on papers that referred to the sperm AD during capacitation, using the same key words, in curated scientific literature databases (PubMed, Scopus, Web of Science). Then, the databases were compared, and a third researcher verified the correctness of the record inserted and resolved eventual conflicts.

The database was realized in Microsoft Excel 2013 (Microsoft Corporation, Albuquerque, NM, USA) and contains the following fields:

- *Molecule involved in biochemical reaction (source)*: molecules participating in the interaction as source;
- *Interaction*: kind of interaction between the molecules or the structures;
- *Molecule or anatomical structure involved in biochemical reaction (target)*: molecules participating to the interaction as target;
- *Alias*: eventual aliases;
- *Role*: physiological and/or pathological role of the molecule/reaction related to fertility;
- *Reference*: article reporting the above mentioned data;
- *Notes*: any further information that could be useful in the study.

Once the databases were created, we obtained the Actin Dynamic Control Network (ADCN).

Once the database was completed and the networks were realized, the main topological parameters (exposed in Table 6) were assessed by using the Network Analyzer plug-in for Cytoscape (open source bioinformatics software platform for visualizing molecular interaction networks and integrating with gene expression profiles and other state data).

### 4.2. Identification of Predicted Interactions with STRING

To identify and predict new molecular interactions of molecules included in we used STRING (Search Tool for the Retrieval of Interacting Genes/Proteins, https://string-db.org/ 06/12/2019) [47]. It is a database that includes known and predicted protein interactions. They could be either direct (physical) or indirect (functional) associations, and are derived from different sources: genomic context, high-throughput experiments, conserved co-expression, and previous knowledge. From the data obtained using STRING a new network was obtained, the Enriched Actin Dynamic Control Network (ADCN_E) by filtering the data for the *Homo sapiens* species and adopting a medium confidence score of 0.400.

### 4.3. Network Exhaustive Analysis and Visualization

All the networks were realized and analyzed using Cytoscape 3.1.2 [48]. The analysis was carried out considering them as directed and assessing the topological parameters listed and described in Table 4.

The identification of bottlenecks within the ADCN was carried out by using the Cytoscape plug-in CytoHubba. It implements the following algorithm for bottleneck calculation: let Ts be a shortest path tree rooted at node s. BN(v) = Σs∈V ps(v) where ps(v) = 1 if more than |V(Ts)|/ 4 paths from node s to other nodes in Ts meet at the vertex v; otherwise ps(v) = 0 [49]. This parameter is a measure of the node centrality expressed as the number of shortest paths in which the node is involved, and is related to the importance of nodes in controlling the information flux within directed networks [50].

We identify the bottleneck of network, and on their basis, we computed the relative network.

## Figures and Tables

**Table 1:** Main Topological Parameters of ADCN_E network. List of the main topological parameters evaluated in the network with their values.

Parameter	DB Network	STRING Network	Merged Network
N of nodes	167	49	188
N of edges	274	287	558
Clustering Coefficient	0.037	0.341	0.127
Diameter	14	5	12
Shortest Path Length	8837 (31%)	436 (19%)	14832 (41%)
Characteristic Path Length	5.760	1.505	5.246
Averaged Number of Neighbors	2.922	11.633	5.471
**In Degree**			
Exponent (γ)	−1.314	−0.608	−1.183
Coefficient of Correlation (r)	0.996	0.734	0.988
Coefficient of Determination (R^2^)	0.910	0.527	0.871
**Out Degree**			
Exponent (γ)	−1.708	−0.675	−1.314
Coefficient of Correlation (r)	0.989	0.935	0.991
Coefficient of Determination (R^2^)	0.885	0.735	0.879
**Node Degree vs. Cluster. Coeff.**			
Coefficient of Determination (R^2^)	0.333	0.085	0.003

**Table 2:** Network hubs. List of the highly connected nodes (hubs) with their corresponding number of edges.

Node Name	Node Degree
AD	36
CDC42	34
PKA	30
SRC	28
CDK1	24
CCNB1	23
CCNA2	22
CDK2	22
PLK1	22
PRC1	21
BUB1	20
BUB1B	20
CCNB2	20
MAD2L1	20
CDC16	20
CDC20	20
CDC23	20
PTTG1	20
AKAP3	19
ANAPC1	19
CCNE1	19
CKS1B	19
ANAPC10	19
BUB3	19
cAMP	19
NEK2	18
[Ca2+]i	15
ACTA1	15
CDKN1B	15
MAPK1	15
RhoA	13

**Table 3:** The two different hubs subpopulations, based on node clustering coefficient.

	Node Name	Clustering Coefficient
**Subpopulation 1**	CCNE1	0,4737
	ANAPC1	0,4708
	CKS1B	0,4708
	ANAPC10	0,4708
	CCNB2	0,4684
	CDC16	0,4684
	CDC23	0,4684
	BUB3	0,4591
	BUB1	0,4579
	BUB1B	0,4579
	MAD2L1	0,4579
	CDC20	0,4579
	PTTG1	0,4474
	NEK2	0,4444
	CCNA2	0,4048
	PLK1	0,4048
	CDKN1B	0,3952
	CCNB1	0,3893
	PRC1	0,3789
	CDK2	0,3788
	CDK1	0,3775
**Subpopulation 2**	MAPK1	0,1429
	ACTA1	0,1346
	SRC	0,1000
	CDC42	0,0860
	[Ca2+]i	0,0758
	RhoA	0,0641
	PKA	0,0498
	AKAP3	0,0381
	AD	0,0331
	cAMP	0,0286

**Table 4:** Nodes and bottleneck score. The table shows the first thirty-one nodes with the bottleneck score for each node.

Name	Bottleneck Score
PKA	81
CDC42	59
AD	57
SRC	40
p-Tyr	22
cAMP	16
RhoA	14
PIP3	12
LIMK	11
Wasp	11
Arp2/3	11
ROS	8
AKAP3	8
PP2A	8
CDK1	8
PIP2 cleavage	7
PLK1	7
PLD	6
RAF1	6
CAMKII	5
[Ca2+]i	5
PI4P	5
sAC	5
AKAP4	5
PIP2	4
proAKAP4	4
MAPK1	4
RhoA GTPase	4
HCO3-	4
CAV1	3
LPAR-LPA	3

**Table 5:** Role of MN controllers in mammalian sperm physiology, where known.

Node Name	Node Degree	Bottleneck Score	Function (with Focus on Mammalian Spermatozoa Physiology)
AD	36	82	
PKA	30	88	Key effector of the bicarbonate-dependent cAMP/protein kinase A (PKA) pathway that leads to the control of p-Tyr of sperm proteins during capacitation. Its activation is correlated to a myriad of biochemical events.
CDC42	34	34	Controller of cell cycle, controller of sperm AD.
SRC	28	23	A non-receptor tyrosine kinase protein that in humans is encoded by the SRC gene. This protein phosphorylates specific tyrosine residues in other tyrosine kinases. An elevated level of activity of c-Src tyrosine kinase is suggested to be linked to cancer progression by promoting other signals. Mutations in this gene could be involved in the malignant progression of colon cancer.
CCNA2	22	14	Controller of cell cycle, controller of sperm AD.
cAMP	19	13	Second messenger of the bicarbonate-dependent cAMP/protein kinase A (PKA) pathway.
AKAP3	19	13	It is expressed in spermatozoa and localized to the acrosomal region of the sperm head, as well as the length of the principal piece. It may function as a regulator of motility, capacitation, and the acrosome reaction (AR)
CDK1	24	6	Controller of cell cycle, controller of sperm AD.
ACTA1	15	15	Polymerizes and depolymerizes during capacitation.
BUB1	20	7	It plays a key role in the establishment of the mitotic spindle checkpoint and chromosome congression.
[Ca2+]i	15	12	Second messenger involved in virtually all the biochemical event related to the capacitation.
RhoA	13	12	It interacts with proteins involved in capacitation and the AR, and RhoA signaling in sperm may be targeted by AKAPs.

**Table 6:** Main topological parameters assessed. The twelve main topological parameters that have been examined are defined.

Parameter	Definition
Connected Components	Number of networks in which any two vertices are connected to each other by links, and which is connected to no additional vertices in the network.
Number of nodes	Total number of molecules involved.
Number of edges	Total number of interactions found.
Clustering coefficient	Calculated as *C*I = 2*n*I/*k*I(*k*I–1), where *n*I is the number of links connecting the *k*I neighbors of node I to each other. It is a measure of how the nodes tend to form clusters.
Network diameter	The longest of all the calculated shortest paths in a network.
Shortest paths	The length of the shortest path between two nodes *n* and *m* is *L* (*n*, *m*). The shortest path length distribution gives the number of node pairs (*n*, *m*) with *L*(*n*,*m*) = *k* for *k = 1*,*2*,…
Characteristic path length	Expected distance between two connected nodes.
Averaged number of neighbors	Mean number of connections of each node.
Node degree	It is the number of interaction of each node.
Node degree distribution	It represents the probability that a selected node has *k* links.
γ	Exponent of node degree equation.
R^2^	Coefficient of determination of node degree vs. number of nodes, on logarithmized data.

## Supplementary Materials

Supplementary materials can be found at https://www.mdpi.com/1422-0067/20/17/4236/s1.
